# Longitudinal Changes in Kinesiophobia, Psychological Readiness, and Knee Function Across Anterior Cruciate Ligament Reconstruction Rehabilitation Phases

**DOI:** 10.3390/healthcare14070879

**Published:** 2026-03-29

**Authors:** Abdullah H. AlMuhaya, Mai Aldera, Dalia M. Alimam

**Affiliations:** 1 Joint Clinics, Medical Rehabilitation Center, Riyadh 13515, Saudi Arabia; 2 Department of Health Rehabilitation Sciences, College of Applied Medical Sciences, King Saud University, Riyadh 11451, Saudi Arabia; maldera@ksu.edu.sa (M.A.); dalimam@ksu.edu.sa (D.M.A.)

**Keywords:** anterior cruciate ligament reconstruction, kinesiophobia, psychological readiness, return to sport, rehabilitation, longitudinal study

## Abstract

**Background/Objectives:** Anterior cruciate ligament reconstruction (ACLR) is a common orthopedic procedure; however, successful return to sport (RTS) remains a major challenge influenced by both physical and psychological factors. Kinesiophobia and psychological readiness are crucial yet inadequately studied components of rehabilitation that may change across distinct phases. This study aimed to examine longitudinal, phase-specific changes in kinesiophobia, psychological readiness, and patient-reported knee function across standardized ACLR rehabilitation phases. **Methods:** A retrospective longitudinal cohort design was employed. Data were extracted from 45 patients who completed ACLR rehabilitation at a specialized musculoskeletal center in Riyadh, Saudi Arabia. Participants were assessed across four rehabilitation phases: Phase One (0–1 month), Phase Two (>1–3 months), Phase Three (>3–6 months), and Phase Four (>6 months post-ACLR). Outcomes included the Tampa Scale of Kinesiophobia (TSK-17), the ACL–Return to Sport after Injury scale (ACL-RSI), and the International Knee Documentation Committee subjective knee form (IKDC), administered using validated Arabic versions. Linear mixed-effects models with Bonferroni-adjusted pairwise comparisons were used to evaluate phase-related changes. **Results:** Significant fixed effects of rehabilitation phase were observed for all outcomes (*p* < 0.001). Kinesiophobia declined substantially from Phase One (mean 51.5) to Phase Three (34.7), with the greatest reduction between Phases Two and Three, followed by stabilization in Phase Four. Psychological readiness increased progressively across all phases (ACL-RSI: 37.1 to 61.8). Knee function demonstrated the greatest improvement during late rehabilitation (IKDC: 37.6 to 75.8). **Conclusions:** Psychological and functional recovery following ACLR follow distinct temporal trajectories rather than improving synchronously. Kinesiophobia declines most markedly during mid-rehabilitation, while functional gains peak in late rehabilitation. These findings support integrating structured psychological screening into phase-specific ACLR rehabilitation protocols.

## 1. Introduction

Anterior cruciate ligament (ACL) rupture is one of the most common and serious injuries in sports medicine, with an estimated global incidence exceeding two million cases annually [1]. It predominantly affects young, physically active individuals and is associated with prolonged absence from sport, high healthcare costs, and an increased long-term risk of knee osteoarthritis [2,3]. Anterior cruciate ligament reconstruction (ACLR) is considered the standard surgical treatment to restore mechanical knee stability and facilitate return to sport (RTS) [4].

Despite advances in surgical techniques and rehabilitation protocols, RTS outcomes remain suboptimal [4]. Only approximately 55–65% of athletes return to their pre-injury level within two years after ACLR [4,5]. This discrepancy between objective recovery and successful reintegration into sport has shifted attention toward psychological determinants of rehabilitation outcomes.

Among these determinants, kinesiophobia and psychological readiness have emerged as key constructs [5]. Kinesiophobia refers to an excessive fear of movement or reinjury that persists despite adequate tissue healing [6]. Psychological readiness, in contrast, encompasses confidence, emotional responses, and risk appraisal related to RTS [7]. Elevated fear has been associated with reduced participation in progressive loading and sport-specific activities, whereas low readiness is linked to delayed RTS and lower return rates [8,9]. A systematic review by Nwachukwu et al. demonstrated that psychological factors account for a significant proportion of failure to RTS following ACLR, independent of physical recovery [10].

Several theoretical frameworks inform this relationship, including the Fear-Avoidance Model [6], the Biopsychosocial Model [11], and Self-Efficacy Theory [12,13], each highlighting distinct mechanisms through which psychological factors shape rehabilitation engagement and recovery outcomes.

Contemporary RTS frameworks advocate multidimensional decision-making, integrating psychological readiness with objective measures of strength, function, and biomechanics [14]. Empirical evidence supports the negative influence of kinesiophobia and the positive role of readiness in RTS outcomes [7,15]. However, most studies employ cross-sectional designs or assess psychological variables at isolated time points, limiting our understanding of how these constructs evolve during rehabilitation [16]. Furthermore, psychological and functional outcomes are often examined independently, reducing insight into their temporal interaction [17]. Re-injury risk and long-term RTS outcomes remain a significant concern, particularly among amateur athletes [18,19].

Although psychological factors are recognized as central to ACLR recovery, longitudinal phase-based investigations remain limited. Early studies focused on fear of reinjury, often using cross-sectional approaches [20]. Later research emphasized psychological readiness, measured through the ACL-RSI, but typically assessed it in isolation or at widely spaced post-operative time points [5]. Studies combining psychological and functional measures have frequently relied on single-time assessments, restricting the evaluation of temporal trajectories [21].

While several investigations have explored psychological changes during recovery [5,13,20], fewer have aligned assessments with clearly defined, clinically meaningful rehabilitation phases [16,21]. Preoperative psychological factors have also been identified as modifiable predictors of return to physical activity following ACLR [22]. Moreover, most evidence originates from Western populations, with minimal representation of Arabic-speaking cohorts, despite the availability of validated Arabic versions of the TSK-17, ACL-RSI, and IKDC [23,24,25]. This gap is clinically meaningful, as formal psychological support is rarely integrated into rehabilitation pathways in Arabic-speaking healthcare contexts, making phase-specific psychological monitoring particularly relevant for this population. This limited representation restricts the development of contextually informed rehabilitation strategies and reduces broader applicability across diverse populations.

To our knowledge, few studies have simultaneously examined longitudinal changes in kinesiophobia, psychological readiness, and patient-reported knee function within a single cohort using a clearly phase-based rehabilitation framework. Mapping these trajectories may help identify periods of psychological vulnerability and inform optimized intervention timing.

This study aimed to retrospectively examine longitudinal changes in kinesiophobia, psychological readiness, and patient-reported knee function across predefined rehabilitation phases following ACLR. Kinesiophobia (TSK-17) was designated the primary outcome, while psychological readiness (ACL-RSI) and knee function (IKDC) were evaluated as secondary outcomes.

Three hypotheses were formulated: (1) Kinesiophobia will be the highest in the earliest rehabilitation phase and will decrease progressively across subsequent phases. (2) Psychological readiness will be the lowest in the earliest rehabilitation phase and will increase progressively across subsequent phases. (3) Patient-reported knee function will be lowest in the earliest rehabilitation phase and will improve progressively across subsequent phases.

## 2. Methodology

A retrospective longitudinal cohort design was adopted to examine changes in kinesiophobia, psychological readiness, and self-reported knee function across predefined phases of ACLR rehabilitation. This design enabled evaluation of real-world rehabilitation trajectories using routinely collected clinical data without altering standard care delivery. This approach was selected due to the feasibility of accessing comprehensive longitudinal records and ethical considerations related to minimizing patient burden. Given the exploratory objective of mapping phase-specific psychological and functional changes, a retrospective design was considered appropriate to generate clinically meaningful insights that may inform future prospective and interventional studies.

### 2.1. Population and Sampling

Data were retrospectively extracted from the electronic medical records of patients treated at Joint Clinics, a private musculoskeletal and sports rehabilitation center in Riyadh, Saudi Arabia. Records were reviewed for the period spanning 5 September 2024, to 1 October 2025. The clinic primarily manages post-operative rehabilitation for physically active individuals and recreational athletes (defined as persons engaging in structured physical activity or organized sport at least twice weekly, independent of competitive level) and receives referrals from board-certified orthopedic surgeons with subspecialty training in knee ligament surgery following ACLR.

**Figure 1:**
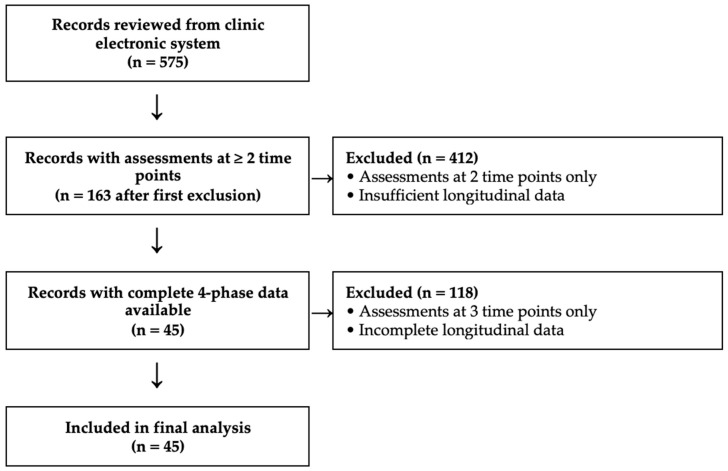
Flow diagram illustrating the screening and inclusion process.

Eligible participants were identified according to predefined criteria, independent of sex, sport type, or competitive level. A total of 575 patient records were initially reviewed. Of these, 412 had assessments at only two time points and were excluded, 118 had assessments at three time points and were excluded, and the first 45 consecutive records containing complete data across all four rehabilitation phases were included. Recruitment was stopped at 45 to remain consistent with the a priori sample size estimate of 44 participants. The screening and inclusion process is illustrated in Figure 1.

Patients were categorized into four rehabilitation phases based on time since surgery: Phase One (0–1 month), Phase Two (>1–3 months), Phase Three (>3–6 months), and Phase Four (>6 months, concluding at approximately 9 months at formal RTS clearance). Although time windows defined phase boundaries, progression was criteria-based; patients advanced only upon meeting objective thresholds including pain levels, range of motion, limb symmetry indices, strength, and functional performance, per the clinic’s structured ACLR protocol. Variability within Phase Four reflects individual differences in criteria attainment. All Phase Four assessments were conducted at the formal RTS clearance visit, standardizing the evaluation point within this phase. The full rehabilitation protocol, including phase-specific objectives and progression criteria, is provided as Supplementary Materials.

### 2.2. Inclusion and Exclusion Criteria

Patients were included if they were aged 18 years or older, with no upper age limit applied, had undergone isolated ACLR regardless of graft type, and possessed documented rehabilitation records containing assessments across all four predefined rehabilitation phases for all three outcome measures: the Tampa Scale of Kinesiophobia (TSK-17), the ACL–Return to Sport after Injury (ACL-RSI) scale, and the International Knee Documentation Committee subjective knee form (IKDC). No BMI limit was applied; BMI was recorded as a demographic variable only. Inclusion was limited to patients able to read and communicate in Arabic, as validated Arabic versions of all instruments were administered.

Exclusion criteria included concomitant knee surgical procedures other than isolated ACLR, concomitant meniscal pathology requiring surgical treatment, incomplete medical records lacking any of the required outcome measures, or failure to complete assessments across all four rehabilitation phases.

### 2.3. Outcome Measures

Validated Arabic versions of patient-reported outcome measures were used to assess psychological and functional domains.

TSK-17: Kinesiophobia was measured using the 17-item Tampa Scale of Kinesiophobia [26]. Items are rated on a four-point Likert scale, yielding total scores ranging from 17 to 68, with higher scores indicating greater fear of movement or reinjury. It has been suggested that a score above 37 reflects high levels of kinesiophobia. The validated Arabic version was used [23,27].

ACL-RSI: Psychological readiness to RTS was assessed using the 12-item ACL–Return to Sport after Injury scale [7]. Scores range from 0 to 100 and reflect emotional response, confidence, and perceived risk related to RTS. Higher scores indicate greater readiness; values ≥ 56 have been associated with successful RTS, while scores ≥ 76 are linked with return to pre-injury performance levels [5]. The validated Arabic version was administered [24].

IKDC: Self-reported knee function was evaluated using the IKDC [28]. This instrument consists of 18 items assessing symptoms, functional limitations, and sport participation. Scores range from 0 to 100, with higher scores indicating better function [29]. The validated Arabic version was used [25].

In early rehabilitation phases, TSK-17 and ACL-RSI scores were interpreted as indicators of psychological adaptation rather than definitive measures of RTS readiness.

### 2.4. Data Collection

Data extraction was performed by authorized members of the research team with formal access to the clinic’s electronic system. Assessments were conducted at each rehabilitation phase transition, corresponding to the point at which a patient met the criteria to progress to the next phase. Each patient contributed one assessment per phase, recorded at the clinical visit closest to the phase transition point. In cases where multiple visits occurred within a phase window, the assessment recorded at the phase transition visit was used. Phase Four assessments were conducted at the formal RTS clearance evaluation, typically at approximately nine months post-surgery. Extracted variables included demographics, relevant clinical history, sport type, date of surgery, rehabilitation phase classification, and TSK-17, ACL-RSI, and IKDC scores. None of the authors were involved in the surgical treatment or rehabilitation delivery of the included patients; data were extracted retrospectively from existing clinical records

### 2.5. Ethical Considerations

Ethical approval for the retrospective use of anonymized clinical data was granted by the Research Ethics Committee of the Department of Health and Rehabilitation Sciences, Prince Sattam bin Abdulaziz University (Approval No. RHPT/025/005). All data were anonymized prior to analysis and screened for completeness and internal consistency.

### 2.6. Statistical Analysis

Statistical analyses were conducted using SPSS version 31.0 (IBM Corp., Armonk, NY, USA) was used for all statistical analyses. Figures were generated using Numiqo (https://numiqo.com, accessed on 20 January 2026). Descriptive statistics were calculated for demographic and outcome variables across phases. Linear mixed-effects models were employed to examine within-subject changes in TSK-17, ACL-RSI, and IKDC scores across rehabilitation phases, with rehabilitation phase specified as a fixed effect and participant (ID) included as a random effect. Models were estimated using restricted maximum likelihood (REML), and degrees of freedom were calculated using the Satterthwaite approximation. An unstructured covariance matrix was specified to account for within-subject correlations across phases. Significant main effects were followed by Bonferroni-adjusted pairwise comparisons of estimated marginal means. Mean differences with 95% confidence intervals are reported as indicators of effect magnitude for pairwise comparisons. Standardized effect sizes (Cohen’s d) were additionally calculated for each pairwise phase comparison to enhance interpretation of clinical relevance. Model residuals were inspected to assess assumptions of normality and homoscedasticity. Statistical significance was set at *p* < 0.05. Only participants with complete data across all phases were included; no imputation was performed.

### 2.7. Sample Size Calculation

A priori power analysis was conducted using G*Power 3.1 based on a repeated-measures ANOVA with four measurement time points. Although the primary analyses were performed using linear mixed-effects models, the repeated-measures ANOVA approach was used for sample size estimation due to the absence of widely implemented closed-form solutions for power calculation in mixed models. Based on prior findings suggesting minor responsiveness to the TSK in ACL populations [30], an effect size of Cohen’s f = 0.20 was adopted. Parameters included α = 0.05, power = 0.80, an assumed correlation between repeated measures of 0.5, and a nonsphericity correction (ε) of 0.75. The analysis indicated that a minimum of 44 participants would be required to detect statistically significant changes in kinesiophobia across rehabilitation phases.

## 3. Results

### 3.1. Participant Characteristics

A total of 45 participants who underwent ACLR completed all four assessment time points and were included in the analysis. The cohort consisted mostly of males (*n* = 37, 82.2%), with a mean age of 27.1 ± 6.9 years. The left knee was more frequently injured (*n* = 31, 68.9%) than the right knee. Hamstring autograft was the most used graft type (*n* = 21, 46.7%), followed by quadriceps tendon (*n* = 14, 31.1%) and bone–patellar tendon–bone grafts (*n* = 10, 22.2%).

Regarding primary sporting activity, gym-based training was reported most frequently (*n* = 15, 33.3%), while football, recreational sports, and running were each reported by 20.0% of participants (*n* = 9 each); basketball was the least represented activity (*n* = 3, 6.7%). At 9 months post-surgery, most participants (*n* = 36, 80.0%) were cleared to RTS, while 20.0% were not (*n* = 9, 20.0%). Detailed demographic and clinical characteristics are presented in Table 1.

### 3.2. Outcome Score Distribution Across Rehabilitation Phases

**Figure 2:**
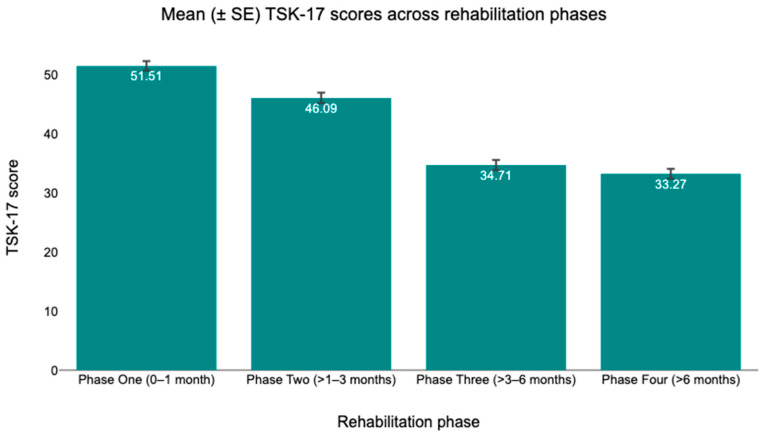
Mean (±SE) TSK-17 scores across rehabilitation phases.

**Figure 3:**
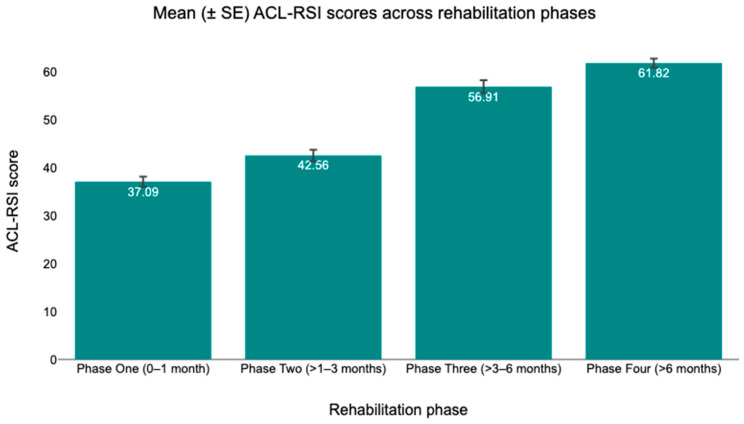
Mean (±SE) ACL-RSI scores across rehabilitation phases.

**Figure 4:**
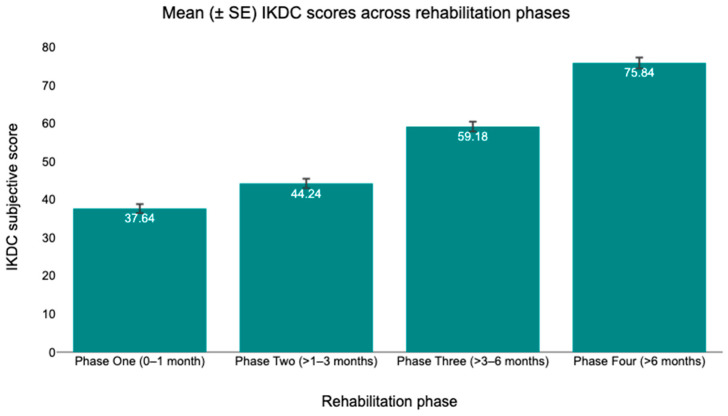
Mean (±SE) IKDC scores across rehabilitation phases.

**Figure A1:**
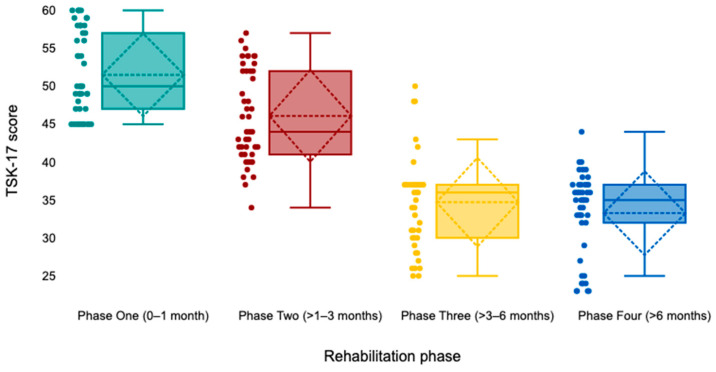
Distribution of individual TSK-17 scores across rehabilitation phases.

**Figure A2:**
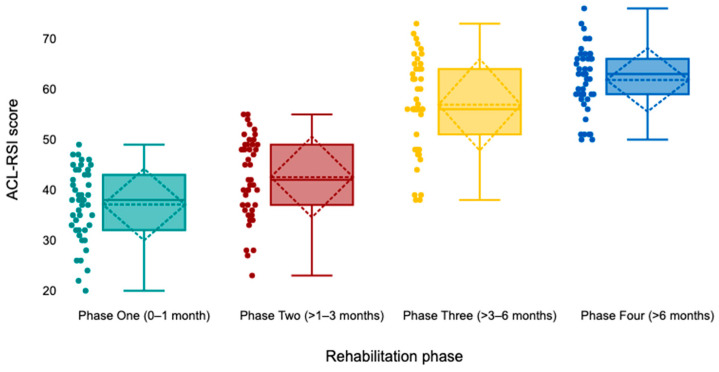
Distribution of individual ACL-RSI scores across rehabilitation phases.

**Figure A3:**
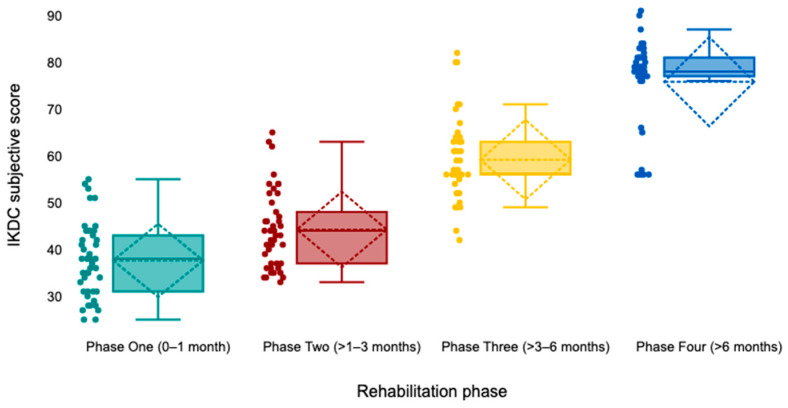
Distribution of individual IKDC scores across rehabilitation phases.

Table 2 presents the mean (SD) values of TSK-17, ACL-RSI, and IKDC scores across the four rehabilitation phases following ACLR. Average TSK-17 scores decreased across successive phases, from 51.5 ± 5.5 in Phase One to 46.1 ± 6.1 in Phase Two, followed by a further reduction to 34.7 ± 5.9 in Phase Three and 33.23 ± 5.6 in Phase Four. ACL-RSI scores increased progressively over time, with mean values of 371 ± 7.2 in Phase One, 42.6 ± 8.1 in Phase Two, 56.9 ± 9.2 in Phase Three, and 61.8 ± 6.3 in Phase Four. Similarly, IKDC scores demonstrated a consistent increase across rehabilitation phases, rising from 37.6 ± 7.9 in Phase One to 44.2 ± 8.2 in Phase Two, 59.2 ± 8.6 in Phase Three, and reaching 75.8 ± 9.6 in Phase Four. Overall, all outcome measures exhibited systematic changes across the rehabilitation timeline, with decreasing TSK-17 scores and increasing ACL-RSI and IKDC scores from early to late rehabilitation phases. Mean changes in outcome scores across rehabilitation phases are illustrated in Figure 2 (TSK-17), Figure 3 (ACL-RSI), and Figure 4 (IKDC). To provide additional transparency concerning within-phase variability and individual-level dispersion, distributions of individual scores for each outcome across rehabilitation phases are presented in the Appendix A (Figure A1, Figure A2 and Figure A3).

### 3.3. Linear Mixed Model Analysis

Linear mixed model analysis demonstrated a statistically significant main effect of rehabilitation phases on all outcome measures (Table 3). For kinesiophobia (TSK-17), the effect of rehabilitation phases was significant, with *F*(3, 44) = 216.4, *p* < 0.001. Psychological readiness (ACL-RSI) also showed a significant main effect of rehabilitation phases, *F*(3, 44) = 233.8, *p* < 0.001. Similarly, knee function (IKDC) demonstrated a significant effect of phase, with *F*(3, 44) = 348.4 and *p* < 0.001.

Pairwise comparisons of estimated marginal means were conducted to identify between-phase differences across rehabilitation (Table 4). For TSK-17, significant reductions were observed between Phase One and Phase Two, Phase One and Phase Three, and Phase One and Phase Four (*p* < 0.001). Significant reductions were also observed between Phase Two and Phase Three and between Phase Two and Phase Four (both *p* < 0.001). However, no statistically significant difference was observed between Phase Three and Phase Four (*p* = 0.193).

For ACL-RSI, psychological readiness increased significantly between each consecutive rehabilitation phase, with the greatest improvement observed between Phase Two and Phase Three (*p* < 0.001). IKDC scores demonstrated significant improvements across all phases, with the most pronounced increase occurring between Phase Three and Phase Four (*p* < 0.001).

All pairwise comparisons were adjusted for multiple testing using the Bonferroni correction. Effect sizes were large to very large for most between-phase comparisons across all three outcomes (Table 4), with the exception of the Phase 3 to Phase 4 TSK-17 comparison, which yielded a small effect (d = 0.25), consistent with the non-significant finding and reflecting stabilization of kinesiophobia in late rehabilitation.

## 4. Discussion

**Figure 5:**
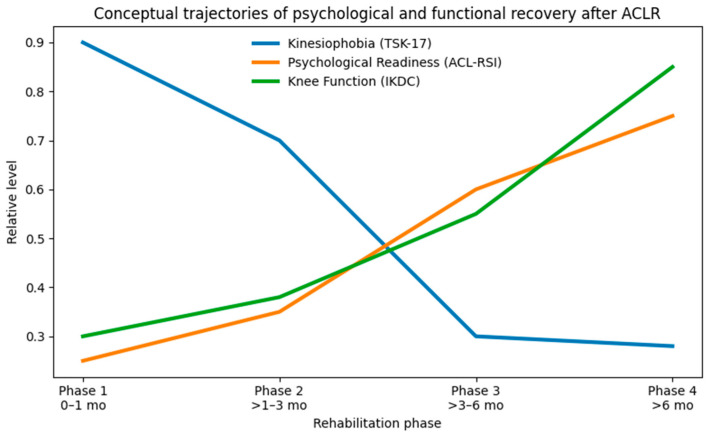
Conceptual illustration of phase-specific changes in TSK-17, ACL-RSI, and IKDC following ACLR. The figure highlights non-synchronous recovery patterns: mid-phase decline in kinesiophobia, progressive increase in readiness, and late-phase improvement in knee function. The figure is intended to provide conceptual interpretation and does not represent raw data. TSK-17: Tampa Scale of Kinesiophobia (17-item); ACL-RSI: Anterior Cruciate Ligament–Return to Sport after Injury scale; IKDC: International Knee Documentation Committee subjective knee form; ACLR: anterior cruciate ligament reconstruction.

Psychological and functional recovery following ACLR were found to follow distinct temporal trajectories rather than improving synchronously across rehabilitation phases. Kinesiophobia declined most markedly during mid-rehabilitation, psychological readiness increased progressively across all phases, and peak improvements in knee function occurred during late rehabilitation. Psychological and functional limitations were most pronounced in the early post-operative period [15,20], yet specific phases emerged as critical windows for meaningful adaptation. These patterns reinforce prior observations that ACLR recovery is phase-dependent rather than uniformly progressive [16,21,31]. These phase-based trends are illustrated conceptually in Figure 5.

### 4.1. Kinesiophobia Across Rehabilitation Phases

Kinesiophobia exhibited a phase-dependent pattern, with the most pronounced reduction occurring between Phase Two (>1–3 months) and Phase Three (>3–6 months). While the primary hypothesis anticipated higher fear in early phases followed by a progressive decline, the data clarify that fear reduction is not evenly distributed over time. Instead, the most substantial and clinically meaningful decline occurs during the transition from early-to-mid rehabilitation (Phase Two to Phase Three), prior to late-stage rehabilitation and formal RTS decision-making [8,10,20].

Elevated TSK-17 scores in early rehabilitation likely reflect heightened threat perception and protective behavioral responses. In this stage, patients have limited experiential evidence to support trust in the reconstructed knee. Movement is often cautious, and avoidance behaviors may be reinforced by uncertainty regarding graft integrity or pain interpretation [15,20].

The sharp decline observed during mid-rehabilitation coincides with structured progression toward unilateral loading, increased strength demands, and early plyometric exposure [15]. These tasks require patients to actively challenge prior beliefs about movement safety. From a Fear-Avoidance Model perspective, this period represents graded exposure through which catastrophic expectations are systematically disconfirmed [6]. Successful task completion without reinjury recalibrates perceived threat and weakens fear-based avoidance patterns [26].

Interestingly, reductions in kinesiophobia plateaued during later phases. This suggests that once core movement-related fear diminishes, residual psychological barriers may relate more to contextual or performance-based anxieties than to a fundamental fear of basic knee loading [30]. The existing cross-sectional literature is refined by these findings, which identify when fear appears most responsive to rehabilitation exposure rather than simply documenting its presence [8,15].

Overall, kinesiophobia emerges as a time-sensitive construct requiring particular attention during early-to-mid-rehabilitation. Structured monitoring during this window may optimize engagement and facilitate downstream psychological adaptation.

### 4.2. Psychological Readiness

In contrast to the non-linear fear trajectory, psychological readiness demonstrated a progressive and largely linear increase across phases. This pattern suggests that readiness develops cumulatively rather than abruptly [4,32].

Low ACL-RSI scores in the early post-operation stage reflect limited confidence and heightened uncertainty. Physical restrictions at this stage constrain opportunities for mastery experiences, which are central to readiness development [13]. As rehabilitation advances and patients achieve incremental milestones—restored range of motion, strength gains, and symmetry in hop testing—confidence builds gradually.

This trajectory aligns closely with Self-Efficacy Theory [12]. Readiness appears to represent the accumulation of successful performance experiences reinforced by clinician feedback and symptom reinterpretation. Rather than reflecting a binary clearance threshold, psychological readiness functions as an evolving synthesis of emotional regulation, confidence, and perceived capability [14]. Importantly, readiness continued to improve even after kinesiophobia reductions stabilized, this finding is consistent with cross-sectional evidence demonstrating associations between physical function and psychological readiness following ACLR [33] and with research linking jumping performance to psychological readiness at six months post-surgery [34]. This temporal dissociation suggests that fear reduction appears to precede continued improvements in readiness, although no causal relationship can be inferred. Once early fear levels decrease, subsequent increases in readiness may be associated with higher-level task mastery and perceived performance competence [19,35].

### 4.3. Knee Function

Patient-reported knee function improved progressively across phases, with the largest magnitude of change observed in late rehabilitation (approximately 6–9 months post-operatively). This finding supports the hypothesis that functional recovery lags early psychological adaptation and aligns with established rehabilitation timelines [17,36].

Early-phase IKDC scores reflect expected limitations associated with graft protection, swelling, and movement restriction [28]. Functional expression is intentionally constrained during this period. As rehabilitation progresses toward high-load, velocity-based, and sport-specific activities, patient-perceived function increases more substantially [37].

Notably, peak functional gains occurred after reductions in fear and alongside increasing readiness. This temporal ordering is consistent with previous research suggesting that psychological adaptation may be associated with greater engagement in demanding physical tasks required for late-stage functional improvements [8,16]. Prior work has demonstrated that unresolved fear or low confidence restricts functional performance despite adequate strength [8,16]. The present findings are consistent with that proposed interaction.

Functional recovery, therefore, may reflect not only biological healing but also prior psychological adaptation, consistent with criteria-based progression models emphasizing integrated psychological and physical readiness [14].

### 4.4. Theoretical Implications

Existing theoretical models are extended by these findings through the introduction of temporal specificity into biopsychosocial recovery frameworks [11,14]. Rather than improving in parallel, psychological and functional domains appear to evolve sequentially, suggesting a potentially interactive pattern that warrants formal testing in future research.

The mid-phase decline in kinesiophobia is consistent with the Fear-Avoidance Model and may be interpreted within its framework [6]. The linear rise in readiness aligns with Self-Efficacy Theory [12]. Finally, the delayed functional peak reinforces integrated RTS frameworks emphasizing multidimensional clearance criteria [14].

Collectively, recovery after ACLR appears neither linear nor purely biomedical. It reflects the dynamic interplay between fear reduction, confidence accumulation, and physical capacity development.

### 4.5. Clinical Implications

Several clinical implications emerge. First, routine psychological monitoring using validated Arabic versions of the TSK-17 and ACL-RSI should be integrated at key milestones [21,23]. Psychological barriers may otherwise go undetected during physical assessments.

Second, the 2–6-month period represents a critical window for fear reduction. The observed temporal dissociation between kinesiophobia stabilization and continued readiness improvement suggests that psychological intervention should not cease once fear levels decline. Clinicians may consider implementing exposure-based therapies and mastery-oriented tasks during mid-to-late rehabilitation to bridge the gap between fear reduction and full psychological readiness for RTS. Structured progression through increasingly challenging sport-specific tasks may support confidence accumulation during this critical period [6].

Third, reassessment around six months should incorporate psychological and functional criteria before progressing to high-load and sport-specific activities [14,37].

Within Arabic-speaking contexts, the availability of culturally validated measures enables structured, evidence-based incorporation of psychological constructs into rehabilitation pathways.

### 4.6. Limitations

Several limitations warrant consideration. The sample consisted predominantly of young male recreational athletes, limiting the generalizability to female or older populations. Additionally, the moderate sample size of 45 participants, while meeting the a priori power estimate, may limit statistical precision and the generalizability of findings to broader ACLR populations. The single-center design may restrict external validity. Retrospective data extraction limits the control over assessment timing. Although the unequally spaced phase intervals are accounted for by the linear mixed model framework, they may still influence the precision of between-phase estimates. The complete-case approach, without imputation, may introduce selection bias if data were not missing completely at random. Outcome measures were exclusively patient-reported, and objective functional measures were not included. Finally, the absence of a control group precludes causal inference.

### 4.7. Future Directions

Future research should adopt prospective, multi-center designs with more diverse samples. Integration of objective biomechanical and strength measures alongside psychological assessments would clarify mechanistic relationships. Randomized controlled trials targeting early kinesiophobia or mid-phase readiness may determine whether modifying psychological variables accelerates functional recovery. Long-term follow-up is needed to assess reinjury rates and sustained participation.

## 5. Conclusions

This longitudinal study provides evidence that individuals undergoing ACLR experience significant, phase-dependent changes in kinesiophobia, psychological readiness, and patient-reported knee function throughout rehabilitation. Importantly, these domains do not improve synchronously. Kinesiophobia declines most markedly during the mid-rehabilitation period, psychological readiness increases progressively across all phases, and peak improvements in knee function occur during late rehabilitation.

These findings are consistent with the interpretation that psychological recovery may not simply reflect passive physical healing but represents a time-sensitive component of rehabilitation that may coincide with functional restoration. Early identification of kinesiophobia and psychological readiness using phase-specific assessment may inform targeted clinical decision-making during rehabilitation.

By highlighting distinct temporal trajectories across psychological and functional domains, these findings support a phase-specific, biopsychosocial approach to ACLR rehabilitation. Conclusions should be interpreted in light of the retrospective design, moderate sample size, unequally spaced phase intervals, and complete-case analytic approach.

## Figures and Tables

**Table 1:** Demographic and clinical characteristics of participants (*n* = 45).

Variable	Category	*n*	%	Mean (SD)	Min	Max	95% CI
Continuous Variables							
Age (years)	—			27.1 (6.9)	18	40	25.1–29.2
BMI (kg/m^2^)	—			25.8 (4.9)	18	35	24.3–27.2
Categorical Variables							
Sex	Male	37	82.2	—	—	—	—
	Female	8	17.8	—	—	—	—
Injured Knee	Left	31	68.9	—	—	—	—
	Right	14	31.1	—	—	—	—
Graft Type	Hamstring autograft	21	46.7	—	—	—	—
	Quadriceps tendon autograft	14	31.1	—	—	—	—
	BTB	10	22.2	—	—	—	—
Primary Sport	Gym	15	33.3	—	—	—	—
	Football	9	20.0	—	—	—	—
	Recreational	9	20.0	—	—	—	—
	Running	9	20.0	—	—	—	—
	Basketball	3	6.7	—	—	—	—
RTS Status at 9 Months	RTS	36	80.0	—	—	—	—
	Non-RTS	9	20.0	—	—	—	—

BMI = body mass index (kg/m^2^); RTS = return to sport; BTB = bone–patellar tendon–bone. Continuous variables are presented as mean (standard deviation); 95% CI = 95% confidence interval of the mean. Categorical variables are presented as frequency (*n*) and percentage. Dashes (—) indicate columns not applicable to that variable type.

**Table 2:** Mean (SD) and 95% Confidence Intervals for TSK-17, ACL-RSI, and IKDC across rehabilitation phases (*n* = 45).

Time Point	TSK-17		ACL-RSI		IKDC	
	Mean (SD)	95% CI	Mean (SD)	95% CI	Mean (SD)	95% CI
Phase One	51.5 (5.5)	49.9–53.2	37.1 (7.2)	34.9–39.3	37.6 (7.9)	35.3–40.0
Phase Two	46.1 (6.1)	44.2–47.9	42.6 (8.1)	40.1–44.9	44.2 (8.2)	41.8–46.7
Phase Three	34.7 (5.9)	32.9–36.5	56.9 (9.2)	54.2–59.7	59.2 (8.6)	56.6–61.8
Phase Four	33.3 (5.6)	31.6–34.9	61.8 (6.3)	59.9–63.7	75.8 (9.6)	72.9–78.7

Data are presented as mean (standard deviation). Phase One: 0–1-month post-ACLR; Phase Two: >1–3 months; Phase Three: >3–6 months; Phase Four: >6 months post-ACLR. TSK-17: Tampa Scale of Kinesiophobia (17-item); ACL-RSI: Anterior Cruciate Ligament–Return to Sport after Injury scale; IKDC: International Knee Documentation Committee subjective knee form.

**Table 3:** Linear mixed model analysis results for TSK-17, ACL-RSI, and IKDC across rehabilitation phases (*n* = 45).

Outcome	F (df1, df2)	*p*
TSK-17	216.4 (3, 44)	<0.001
ACL-RSI	233.8 (3, 44)	<0.001
IKDC	348.4 (3, 44)	<0.001

F: F-statistic; df1: numerator degrees of freedom; df2: denominator degrees of freedom, estimated using the Satterthwaite approximation within the linear mixed model framework. Post hoc pairwise comparisons were adjusted using the Bonferroni correction.

**Table 4:** Bonferroni-adjusted pairwise comparisons of estimated marginal means across rehabilitation phases (*n* = 45).

Outcome	Phase Comparison	Mean Difference (I–J)	95% CI	*p* (Adjusted)	Cohen’s d
TSK-17	Phase 1 vs. Phase 2	5.42	4.56–6.29	<0.001	0.94
	Phase 1 vs. Phase 3	16.80	14.80–18.80	<0.001	2.90
	Phase 1 vs. Phase 4	18.24	15.71–20.78	<0.001	3.15
	Phase 2 vs. Phase 3	11.38	9.50–13.26	<0.001	1.97
	Phase 2 vs. Phase 4	12.82	10.39–15.25	<0.001	2.22
	Phase 3 vs. Phase 4	1.44	−0.36–3.25	0.193	0.25
ACL-RSI	Phase 1 vs. Phase 2	−5.47	−6.60–−4.34	<0.001	0.72
	Phase 1 vs. Phase 3	−19.82	−22.40–−17.25	<0.001	2.40
	Phase 1 vs. Phase 4	−24.73	−27.71–−21.75	<0.001	3.65
	Phase 2 vs. Phase 3	−14.36	−16.76–−11.95	<0.001	1.66
	Phase 2 vs. Phase 4	−19.27	−22.38–−16.15	<0.001	2.65
	Phase 3 vs. Phase 4	−4.91	−7.55–−2.27	<0.001	0.62
IKDC	Phase 1 vs. Phase 2	−6.60	−7.51–−5.69	<0.001	0.82
	Phase 1 vs. Phase 3	−21.53	−23.57–−19.49	<0.001	2.61
	Phase 1 vs. Phase 4	−38.20	−42.34–−34.07	<0.001	4.34
	Phase 2 vs. Phase 3	−14.93	−16.76–−13.10	<0.001	1.78
	Phase 2 vs. Phase 4	−31.60	−35.74–−27.46	<0.001	3.54
	Phase 3 vs. Phase 4	−16.67	−19.92–−13.41	<0.001	1.83

Mean differences are based on estimated marginal means derived from linear mixed models. Mean differences represent Phase I minus Phase J. Positive values indicate higher scores in the earlier phase for TSK-17 and higher scores in the later phase for ACL-RSI and IKDC. *p*-values were adjusted for multiple comparisons using the Bonferroni correction. Cohen’s d interpreted as: small (0.2–0.5), medium (0.5–0.8), large (>0.8). Phase One: 0–1 month; Phase Two: >1–3 months; Phase Three: >3–6 months; Phase Four: >6 months post-ACLR.

## Data Availability

The data supporting the findings of this study are not publicly available due to ethical and privacy restrictions related to patient confidentiality. De-identified data may be made available by the corresponding author upon reasonable request and subject to institutional approval.

## Supplementary Materials

The following supporting information can be downloaded at: https://www.mdpi.com/article/10.3390/healthcare14070879/s1, Table S1: ACLR Phase-Based, Criteria-Driven Rehabilitation Protocol including phase-specific objectives and progression criteria for all four rehabilitation phases.

## Abbreviations

The following abbreviations are used in this manuscript:

ACL	Anterior Cruciate Ligament
ACLR	Anterior Cruciate Ligament Reconstruction
ACL-RSI	ACL–Return to Sport after Injury scale
BMI	Body Mass Index
BTB	Bone–Patellar Tendon–Bone
IKDC	International Knee Documentation Committee subjective knee form
RTS	Return to Sport
TSK-17	Tampa Scale of Kinesiophobia (17-item version)
