# Supplementary material for: Longitudinal Changes in Kinesiophobia, Psychological Readiness, and Knee Function Across Anterior Cruciate Ligament Reconstruction Rehabilitation Phases
Source: Healthcare (Basel). 2026 Mar 29;14(7):879. doi: 10.3390/healthcare14070879 (PMC13073564; doi:10.3390/healthcare14070879)
Supplement: Supplementary file 1 [file healthcare-14-00879-s001.zip › healthcare-4216421-supplementary.pdf]

JOINT CLINICS · RIYADH, SAUDI ARABIA

ACLR Rehabilitation Protocol

Phase-Based, Criteria-Driven Approach · Advance When ALL Criteria Are Met

PHASE 1

Early Rehabilitation

0 – 1 Month

PRIMARY OBJECTIVES

- Protect graft integrity
- Restore ROM & reduce pain/effusion
- Restore quadriceps activation
- Normalize gait pattern

PROGRESSION CRITERIA

- ✓ Effusion ≤ +1 (stroke test)
- ✓ Pain ≤ 2/10 (VAS)
- ✓ Full extension (0°); ≥125° flexion
- ✓ ≥10 SLR without lag
- ✓ Active knee extension ≥5 s hold
- ✓ Independent gait without crutches
- ✓ Stable single-leg stance

PHASE 2

Mid Rehabilitation

1 – 3 Months

PRIMARY OBJECTIVES

- Restore strength & neuromuscular control
- Initiate running readiness
- Controlled plyometric exposure

PROGRESSION CRITERIA

- ✓ Glute bridge ≥25 reps, LSI ≥85%
- ✓ SL bridge & calf raise ≥20 reps, LSI ≥85%
- ✓ SL squat control (90°, 5 reps × 2 s)
- ✓ Balance EO ≥43 s; EC ≥9 s
- ✓ Quad/HS ≥70% LSI; hop ≥70%; full ROM
- ✓ Full squat tolerated; Quad/HS ≥80% LSI

PHASE 3

Late Rehabilitation

3 – 6 Months

PRIMARY OBJECTIVES

- Develop power & landing control
- Agility & change-of-direction (COD)
- Psychological preparation for RTS

PROGRESSION CRITERIA

- ✓ SL raise ≥22 reps, 100% LSI
- ✓ Y-Balance ≥95% LSI
- ✓ Hop battery ≥80% LSI
- ✓ Proper landing mechanics (no dynamic valgus)
- ✓ COD readiness: Quad/HS ≥80% LSI
- ✓ CMJ/DJ ≥80% LSI

PHASE 4

Return to Sport

6 – 9 Months

PRIMARY OBJECTIVES

- Confirm unrestricted RTS readiness
- Physical & biomechanical clearance
- Psychological & load-based readiness

PROGRESSION CRITERIA

- ✓ ACL-RSI ≥70–75
- ✓ TSK-17 ≤37
- ✓ IKDC ≥80–85
- ✓ Hop battery & side hop ≥90% LSI
- ✓ COD/agility ≥90% baseline
- ✓ Isokinetic Quad/HS ≥90% LSI
- ✓ Jump impulse symmetry ≥90%; ACWR 0.8–1.3

ABBREVIATIONS

ACLR = Anterior Cruciate Ligament Reconstruction · ROM = Range of Motion · LSI = Limb Symmetry Index · SL = Single-Leg · SLR = Straight-Leg Raise · VAS = Visual Analogue Scale

EO/EC = Eyes Open / Eyes Closed · HS = Hamstrings · COD = Change of Direction · CMJ = Countermovement Jump · DJ = Drop Jump · ACWR = Acute:Chronic Workload Ratio · RTS = Return to Sport

ACL-RSI = ACL Return to Sport after Injury scale · TSK-17 = Tampa Scale for Kinesiophobia · IKDC = International Knee Documentation Committee Subjective Knee Form
